# Hydroxy Selenomethionine Improves Meat Quality through Optimal Skeletal Metabolism and Functions of Selenoproteins of Pigs under Chronic Heat Stress

**DOI:** 10.3390/antiox10101558

**Published:** 2021-09-29

**Authors:** Yan Liu, Shenggang Yin, Jiayong Tang, Yonggang Liu, Gang Jia, Guangmang Liu, Gang Tian, Xiaoling Chen, Jingyi Cai, Bo Kang, Hua Zhao

**Affiliations:** 1 Key Laboratory for Animal Disease-Resistance Nutrition, Ministry of Education, Animal Nutrition Institute, Sichuan Agricultural University, Chengdu 611130, Sichuan, China; 2018114013@stu.sicau.edu.cn (Y.L.); 2020214004@stu.sicau.edu.cn (S.Y.); 13670@sicau.edu.cn (J.T.); 11988@sicau.edu.cn (G.J.); liugm@sicau.edu.cn (G.L.); 13555@sicau.edu.cn (G.T.); xlchen@sicau.edu.cn (X.C.); 11890@sicau.edu.cn (J.C.); 2 Adisseo Asia Pacific Pte. Ltd., Singapore 188778, Singapore; kevin.liu@adisseo.com; 3 College of Animal Science and Technology, Sichuan Agricultural University, Chengdu 611130, Sichuan, China; bokang@sicau.edu.cn

**Keywords:** selenoprotein, OH-SeMet, chronic heat stress, meat quality, skeletal muscle metabolism, pigs

## Abstract

Chronic heat stress (CHS) induces metabolic changes in skeletal muscle from growth to maintenance that jeopardizes growth performance, carcass traits, and meat quality of pigs. We investigated the protective effect of dietary organic selenium (hydroxy-4-methylselenobutanoic acid, OH-SeMet) on CHS-induced skeletal muscle damages of growing pigs, and the corresponding responses of selenoproteins. A total of 40 ((Landrace ×Yorkshire) × Duroc) pigs with an average live weight of 49.64 ± 2.48 kg were used in this 4-week trial. Pigs were randomly allotted to 5 groups: The control group was raised on a basal diet in a thermoneutral environment (22 ± 2 °C); and four CHS groups were raised on a basal diet and supplemented with Se 0.0, 0.2, 0.4, and 0.6 mg/kg as OH-SeMet, respectively, in hyperthermal condition (33 ± 2 °C). CHS resulted in significant decrease of growth performance, carcass traits, and meat quality, which were associated with reduced (*p* < 0.05) serum alkaline phosphatase (ALP) and total superoxide dismutase (T-SOD) and increased (*p* < 0.05) serum creatine (CK), sarcous heat shock protein 70 (HSP70), glucokinase (GCK), phosphoenolpyruvate carboxykinase (PEPCK), and malondialdehyde (MDA) contents. Meanwhile, four metabolism-related genes and seven selenoprotein encoding genes were abnormally expressed in skeletal muscle. Dietary OH-SeMet addition partially alleviated the negative impact of CHS on carcass traits and improved meat quality. These improvements were accompanied by the increase in Se deposition, the anti-oxidative capacity of serum and muscle, and protein abundance of GPX1, GPX3, GPX4, and SELENOP. Supplementation with 0.6 mg Se/kg (OH-SeMet) restored the sarcous PEPCK, and 0.4 and 0.6 mg Se/kg (OH-SeMet) restored all abnormally expressed metabolism-related and selenoprotein encoding genes. In summary, dietary supplementation with OH-SeMet beyond Se requirement mitigated CHS-induced depression of carcass traits and meat quality of pigs associated with optimal skeletal metabolism, enhanced antioxidant capacity, and regulation of selenoproteins in skeletal muscle of pigs.

## 1. Introduction

As global warming continues, extreme climate events become frequent and common [1]. High ambient temperature is a common hazard that compromises animal welfare as well as their productivity due to heat stress (HS), causing significant economic losses to global livestock production [2]. The HS of animals is generally classified as acute and chronic, and the acute HS lasts from a few hours to days whilst the chronic HS lasts days to weeks [3]. Chronic heat stress (CHS) causes severe lesions of organs and tissues and dysregulation of energy balance and metabolism [4], which results in the decreased quality of livestock products.

Pigs are sensitive to HS as they have thick layers of subcutaneous adipose tissue and lack functional sweat glands to facilitate heat loss via an evaporative pathway from the skin [5]. Pigs reared long-term in hyperthermal conditions typically reduce their feed intake in an attempt to decrease metabolic heat production as an adaptive response to CHS [6], which has implications for growth performance, carcass yield, composition, and intramuscular fat content (IMF) [7]. The deterioration in meat quality caused by CHS is recognized as one of the major economic losses in the swine industry [8], which represents significant loss ameliorated by nutritional manipulation [9]. Except for the decrease in feed intake, hyperthermia also impairs skeletal muscle metabolism, redox status, and eventually cell damage, which may be the major cause of decreased meat quality [5,10].

The micronutrient trace element selenium (Se) is an essential cellular antioxidant. Se supplementation relieves the HS-induced damage in C2C12 cells, IPEC-J2 cells, and hepatic injury in growing pigs by enhancing antioxidant capacity [11,12,13]. Recent studies reported that Se affects the metabolic process of carbohydrates, proteins, and lipids [14,15]. As we know, Se exerts its biological function mainly through selenoproteins [16], and in total 25 porcine selenoprotein genes have been identified [17]. Our previous study indicates that several selenoprotein encoding genes are involved in the remission of CHS-induced metabolic disorder [11]. Although the exact mechanisms remain unclear, studies have observed the alternation of the protein synthesis pathway (mTOR, 4E-BP1, and RPS6/S6), lipogenesis (FOXO1, FAS, ACC1, and SREBP1), and glucose metabolism- (INSR, IRS1, AKT, PCK2, and GCK) related genes are associated with the abnormal expression of GPX1, GPX4, SELENOH, SELENOP, SELENOS, DIO1, and TXNRD1 [14,15,18,19,20,21,22].

Altogether, studies have demonstrated that CHS causes damages to antioxidant capacity and metabolic homeostasis of skeletal muscle of livestock and poultry [23,24]. Supplementation with antioxidant nutrients, such as Se, alleviates various types of stress on domestic animals [7,25,26,27]. However, the interaction of dietary Se concentration and CHS on skeletal muscle metabolic function remains to be elucidated. Pig meat (pork) is one of the most consumed meats in the world, and pork quality under normal and off-normal physiological conditions has attracted keen interests [9]. As hydroxy-4-methylselenobutanoic acid (OH-SeMet) is a new type of organic Se source with high bioavailability and low toxicity [28], we developed the pig CHS model to investigate: (1) the protective effect of OH-SeMet on CHS-induced growth performance, carcass traits, meat quality, skeletal muscle antioxidant capacity, and metabolic homeostasis damage; and (2) the possible metabolic link between the alleviation of CHS and the functions of selenoproteins in the skeletal muscle.

## 2. Materials and Methods

### 2.1. Animals, Experiment Design, and Management

The animal trial was performed following the by-law of animal protection approved by the Animal Care and Use Committee of the Sichuan Agricultural University (Ethics Approval Code: SCAUAC201808-2).

A total of 40 crossbreed castrated boars (Landrace × Yorkshire) × Duroc aged 14 weeks, with an average live weight of 49.64 ± 2.48 kg, were randomly allocated into 5 treatments with 8 replicates per treatment and 1 pig per replicate (*n* = 8). The control group (CON) was fed a basal diet without Se supplementation and raised in a thermoneutral environment (22 ± 2 °C). The following four treatment groups were fed a basal diet supplemented with selenium in the form of 2-hydroxy-4-methylselenobutanoic acid (OH-SeMet, Selisso^®^ Adisseo France S.A.S., Paris, France) at dosage (mg Se/kg diet): 0.0, 0.2, 0.4, and 0.6, and these 4 groups of animals were subjected to CHS (33 ± 2 °C). The timeline for the CHS challenge is set as following—after three days of acclimatization, the temperature of CHS gradually rises above 35 °C. The basal diet was formulated to meet the nutritional requirements of the National Research Council (2012) for growing pigs weighing 50−75 kg (Table 1), which was a standard experiment diet formulation used in our previous study [29]. All pigs had free access to diet and water, and penned individually in an artificial climate chamber, which allowed temperature setting and control. Temperature (T) and relative humidity (RH) were continuously monitored daily inside the climate chamber for the entire experimental period. The temperature humidity index (THI) was calculated according to the method described previously as below [30]. 

THI = T °C − (0.31 − 0.31RH) × (T °C − 14.4). 

T = temperature in °C; 

RH = relative humidity in %/100

Live weight and feed intake were determined at the beginning and end of the trial, and average daily gain (ADG), feed intake (ADFI), and feed conversion ratio (FCR) were calculated.

### 2.2. Carcass Analysis, Blood and Muscle Sample Collection

Blood samples (10 mL) were collected from the jugular vein at d 28 with anticoagulant-free tubes. Then serum was separated after centrifuging at 2500× *g* for 10 min at 4 °C. Six pigs close to the average body weight from each group were slaughtered at day 28 of the trial after overnight fasting and sedated by electrical stunning followed by exsanguination. The carcass weight (CW) was estimated as the weight of the hot eviscerated carcass, which was calculated at 45 min after harvest, multiplied by 0.98 [31]. The length of the carcass was measured as the straight distance from the midpoint of the pubic symphysis to the midpoint of the first cervical vertebra. The backfat thickness was measured at 6 cm from the edge of the split back at the level of the 3rd to 4th last rib using a Hennessy grading probe (Hennessy and Chong, Auckland, New Zealand). The lean meat content and kill-out proportion were estimated according to the following formula:

Estimated lean meat content (%) = 53.41 − 0.786*x* + 0.266*y*

where *x* = fat depth(mm); *y* = muscle depth(mm).

Kill-out proportion (%) = CW/Live weight

The muscle samples of longissimus dorsi (LD) between the 12th and 13th ribs from the left half of the carcass were dissected and rapidly frozen in liquid nitrogen and stored at −80 °C for subsequent analyses.

### 2.3. Meat Quality Analysis

Meat quality was estimated on the right half of the carcass and all parameters were determined following the methods described in our previous studies with modifications [24,32,33]. Briefly, the following characteristics of meat quality were determined in LD at the 13th and 14th rib level: pH and meat color (L*: lightness, a*: redness, and b*: yellowness) measured at 45 min, 24 h, 48 h, 72 h, and 96 h postmortem. The pH values were measured by an insert electrode (pH-Star, Matthäus, Pöttmes, Germany). The L*, a*, and b* values of the LD were determined using Minolta Chromameter (CR-300, Minolta Camera, Osaka, Japan) with a setting of illuminant D65 and 0° viewing angle and subjective color (5-point scale; 1 = pale to 5 = dark red). Two 2.5 cm LD chops were cut from the 10th rib side of the section and trimmed of epimysium and external fat. One chop was weighed and placed in a Whirl-pak bag and suspended for 24 h in a 4 °C cooler then reweighed to determine drip loss. The other chop was cooked in a water bath until a core temperature of 70 °C was reached. After cooling for 90 min at room temperature, 5 cores (1.2-cm diameter) were taken from the slice parallel to muscle fiber direction. Each core was sheared using a WB blade (1.0 mm wide at 4.5 mm/s) on a Texture Analyzer (XT2, Stable Micro Systems Ltd., Godalming, Surrey, UK), and peak shear force was recorded in kilogram. Chops used for Warner–Bratzler shear force determination were weighed before and after cooking to calculate cooking losses.

### 2.4. Selenium Deposition in Muscle and Blood

The total Se concentration in muscle and blood were determined with a hydride generation flame atomic fluorescence spectrometer (AFS-3100, Hai Guang Instrument, Beijing, China) based on the national food safety standard of China (GB 5009.93-2010), and calculated according to the protocol used in the previous study [34].

### 2.5. Serum Biochemistry and Hormone Analyses

Serum alkaline phosphatase (ALP), creatine kinase (CK), and lactate dehydrogenase (LDH) were measured using an automatic biochemistry analyzer (3100, HITACHI, Tokyo, Japan). Serum cortisol (COR) was determined using a radioimmunoassay kit (Beijing North Institute of Biological Technology, China), following the manufacturer’s instructions.

### 2.6. Antioxidant and Metabolic Enzyme Analyses

Skeletal muscle homogenates and total protein content determination were done as previously described by our group [35]. Glutathione peroxidase (GSH-Px), total superoxide dismutase (T-SOD), total antioxidant capability (T-AOC), and malondialdehyde (MDA) of serum and muscle tissue were measured by colorimetric assay using commercial kits (Jiancheng Bioengineering, Nanjing, China). Tissue lactate dehydrogenase (LDH) was determined using a commercial kit (Jiancheng Bioengineering, Nanjing, China). Tissue phosphoenolpyruvate carboxykinase (PEPCK), Glucokinase (GCK), and fatty acid synthase (FAS) were determined by commercial ELISA kits (Meimian, Yancheng, China) according to the manufacturer’s instructions. All measurements were performed in triplicate.

### 2.7. Real-Time qPCR Analyses

Total mRNA isolation and reverse transcription were performed using a commercial reagent kit (Invitrogen, Carlsbad, CA, USA), and the subsequent quantitative real-time PCR (Q-RT-PCR) was carried out by QuantStudio Real-Time PCR system (QuantStudio 6 Flex, Applied Biosystems, Foster City, CA, USA) as described in our previous study [11]. The primer sequences used for the assayed genes of 12 metabolism-related genes, 25 selenoprotein encoding genes, and 2 house-keeping genes (β-ACTIN and GAPDH) were referenced in our previous study and shown in (Supplementary Table S1) [11].

### 2.8. Western Blot Analyses

The skeletal muscle tissues were homogenized with the cell disruption buffer (RIPA lysis Buffer, Beyotime, Shanghai, China), then the total protein concentration was measured using the BCA kit (Jiancheng Bioengineering, Nanjing, China). The subsequent Western blot process was performed as previously described [11,29]. The primary antibodies were used at the following dilutions: HSP70 (1:5000; ab5439; Abcam, Cambridge, UK), GPX1 (1:1000; 616958; Zen BioScience, Chengdu, China), GPX3 (1:2000; sc-58361, Santa Cruz Biotechnology, Santa Cruz, CA, USA), GPX4 (1:2000; 513309, Zen BioScience, Chengdu, China), SELENOP (1:2000; sc-376858, Santa Cruz Biotechnology, Santa Cruz, CA, USA), SELENOS (1:1000, 15591-1-AP, ProteinTech Group, Chicago, IL, USA) and GAPDH (1:5000; 200306-7E4, Zen BioScience, Chengdu, China).

### 2.9. Statistical Analysis

The experiment was performed under a complete random design (CRD) following a one-way structure treatment design. The effect of CHS and different doses of OH-SeMet supplementation was analyzed using PROC MIXED of SAS 9.2 (SAS Institute, 2003). Multiple treatment comparisons followed the Tukey test using the LSMEAN statement of SAS 9.2 (SAS Institute, 2003), and the letter grouping was obtained using pdmix800 macro (Saxton, 1998) [35]. The UNIVARIATE and HOVTEST statement with Shapiro-Wilk W test and Levene’s test were used for the evaluation of normality and homogeneity of variances and outliers excluded using Grubb’s test [36]. Statistical significance was declared when *p* ≤ 0.05 or highly significant at *p* ≤ 0.01, unless otherwise stated.

## 3. Results

### 3.1. Growth Performance and Carcass Traits

**Figure 1:**
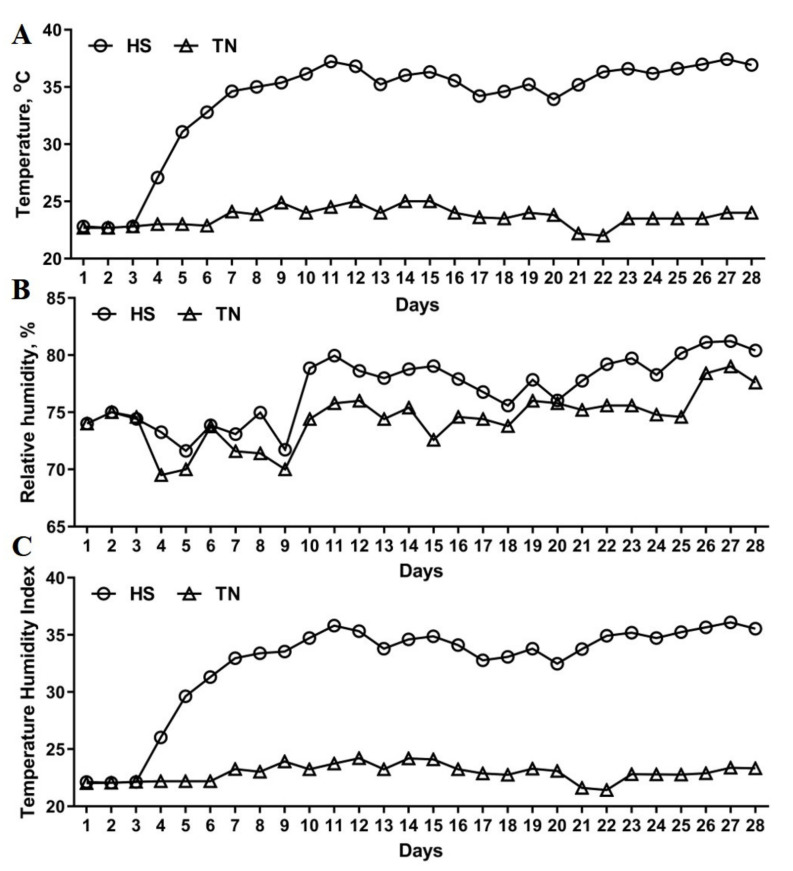
Temperature (°C) (**A**), relative humidity (**B**) and temperature humidity index (**C**) inside the climate chamber. TN, thermoneutral condition; HS, heat stress condition.

The daily recorded Temperature (°C) (Figure 1A) and relative humidity (Figure 1B), and corresponding calculated temperature humidity index (Figure 1C) were shown as Figure 1. For the pigs exposed to hyperthermia environment for 28 days (Figure 1), the CHS led to decreases of ADFI and ADG by 19% and 23% (*p* < 0.05), respectively (Table 2), and the final live weight (LW) by 5.93 kg (*p* < 0.05). Compared to the CHS group, the supplementation of OH-SeMet showed clear trend in improving ADG, final live weight, and FCR, despite the differences not reaching statistical significance (Table 2). As shown in Table 3, CHS decreased (*p* < 0.05) the carcass length, carcass weight and lean proportion of growing pigs. The supplementation with OH-SeMet restored the carcass weight to the control level (*p* > 0.05), while 0.2 mg/kg and 0.4 mg/kg supported carcass length and % lean to control values, respectively (*p* > 0.05). Beyond this, no statistical differences were observed in kill-out, abdominal fat, and eye-muscle area.

### 3.2. Meat Quality and Se Concentration in Serum and Muscle

**Figure 2:**
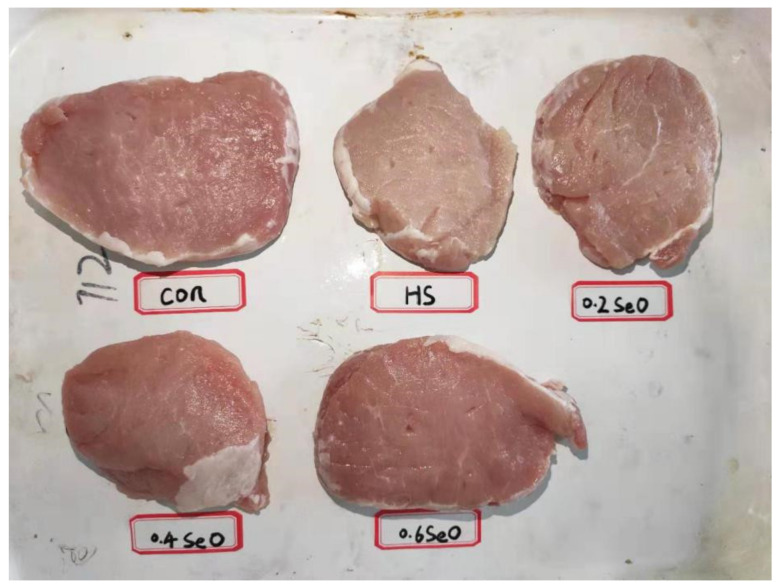
Photographs of representative longissimus dorsi muscle samples 24 h after slaughtering.

**Figure 3:**
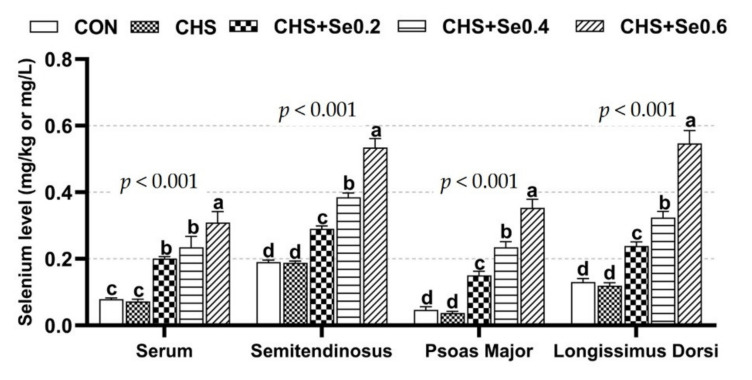
Se concentration in serum and muscle. The results were expressed as Mean ± SEM (*n* = 6). Different alphabet letters (a, b, c, d) denote significant differences (*p* < 0.05).

As shown in Figure 2, CHS had a serious impact on the appearance of pork, which exhibited a pale, not fresh color. These changes were reflected by the alternation of L*, b* and cooking loss of longissimus dorsi muscle (*p* < 0.05) (Table 4). CHS resulted in higher L* 45 min, L* 48 h, L* 72 h, L* 96 h, b* 45 min, b* 24 h, b* 72 h and cooking loss, and numerically lower a* 45 min, a* 24 h, a* 48 h, a* 96h and drop loss. Dietary supplementation with OH-SeMet restored the color of the pork (Figure 2) and alleviated the negative impacts of CHS (Table 4). Supplementation of Se 0.4 and 0.6 mg/kg restored the L* 45 min, L* 24 h, L* 72 h, L* 96 h, b* 45 min, b* 24 h, b* 72 h and cooking loss to the control level (*p* > 0.05), and enhanced (*p* < 0.05) the a* 48 h and a* 72 h. Se supplementation at 0.2 mg/kg also recovered the b* 45 min, b* 24 h and cooking loss (*p* > 0.05). Despite only a tendency (0.05 < *p* < 0.1), dietary supplementation with OH-SeMet limited the drip loss and elevated the pH72h. The analyses of Se in serum and skeletal muscle (Figure 3) suggested CHS exerted no impact on Se concentrations (*p* > 0.05). As expected, the supplementation of OH-SeMet increased Se concentration in serum, semitendinosus, psoas major, and longissimus dorsi muscle (*p* < 0.05).

### 3.3. Antioxidant Enzyme and Malondialdehyde Content of Serum and Muscle

As shown in Table 5, CHS elevated (*p* < 0.05) the malondialdehyde (MDA) content in serum and longissimus dorsi muscle, and decreased the total superoxide dismutase (T-SOD, *p* <0.05) and the total antioxidant capacity (T-AOC, *p* < 0.1) activity in serum. The supplementation with OH-SeMet enhanced the GSH-Px activity in serum (*p* < 0.05), Se supplementation at 0.4 and 0.6 mg/kg increased the GSH-Px activity of longissimus dorsi muscle (*p* < 0.05). The serum T-AOC in all three Se supplementation groups was restored to the control level (*p* > 0.05) and the serum T-SOD was also recovered by Se supplementation at 0.4 and 0.6 mg/kg (*p* > 0.05). Subsequently, dietary supplementation with OH-SeMet kept MDA concentrations at the control level in serum and longissimus dorsi muscle (*p* > 0.05), and no statistical differences were observed in T-SOD and T-AOC activities of the longissimus dorsi muscle.

### 3.4. Serum Biochemical, Hormone, and HSP70 Protein Abundance in Muscle

**Figure 4:**
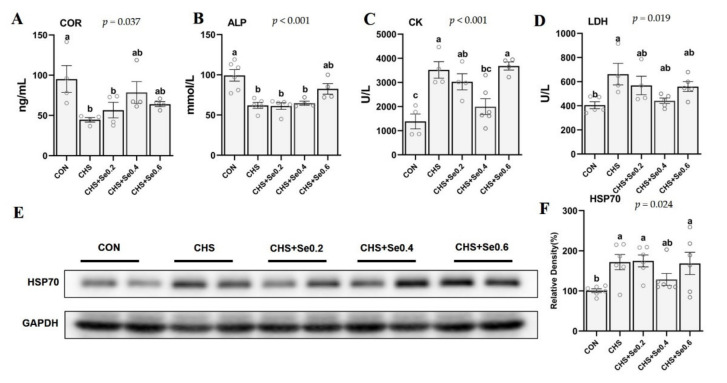
Effects of CHS and OH-SeMet supplementation on serum endocrine (**A**), biochemical parameters (**B**–**D**) and muscle HSP70 protein abundance (**E**, **F**) of growing pigs. COR, cortisol; ALP, alkaline phosphatase; CK, creatine kinase; LDH, lactate dehydrogenase; HSP70, heat shock protein 70. The results are expressed as Mean ± SEM (*n* = 4 or 6); values within the same row not bearing the same superscripts differ (a, b, c, d) (*p* < 0.05).

As shown in Figure 4, CHS up-regulated (*p* < 0.05) the protein abundance of HSP70 in the longissimus dorsi muscle, and supplementation of Se 0.4 mg/kg returned it to the control level (*p* > 0.05) (Figure 4E,F). Meanwhile, CHS led to reduced (*p* < 0.05) serum cortisol (COR) (Figure 4A) and alkaline phosphatase (ALP) (Figure 4B), and increased (*p* < 0.05) serum creatine (CK) (Figure 4C) and lactate dehydrogenase (LDH) (Figure 4D). Dietary Se supplementation also restored the serum LDH to the control level (*p* > 0.05) and Se addition of 0.4 and 0.6 mg/kg recovered the serum COR (*p* > 0.05). The serum ALP concentration was recovered in the 0.6 mg/kg OH-SeMet supplementation group (Figure 4B) (*p* > 0.05) and the serum CK in 0.4 mg/kg OH-SeMet addition group was also decreased to the control level (*p* > 0.05) (Figure 4C).

### 3.5. Metabolism-Related Enzyme Activity and Gene mRNA Expression in Muscle

**Figure 5:**
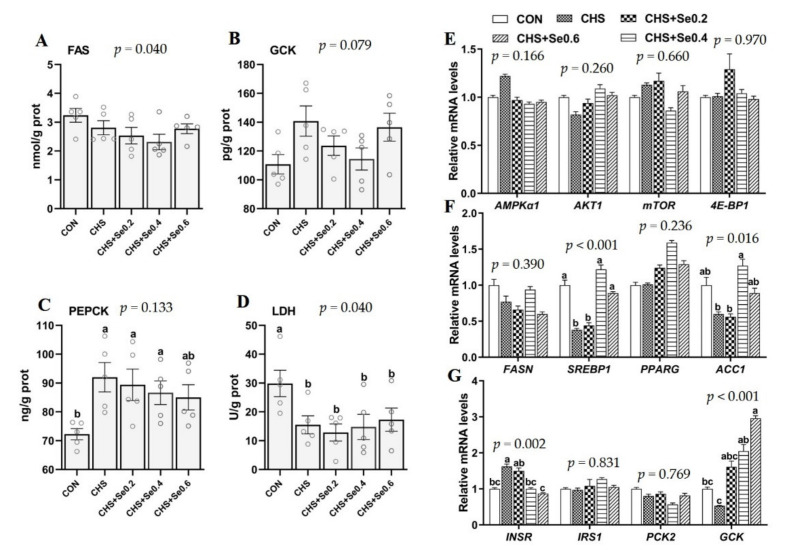
Effects of CHS and Se supplementation on enzyme activity (**A**–**D**) and expression of genes related to protein (**E**), lipid (**F**), and glucose (**G**) metabolism in longissimus dorsi muscle. The results are expressed as Mean ± SEM (*n* = 4 or 6). Different letters (a, b, c, d) denote significant differences (*p* < 0.05).

As shown in Figure 5A–D, CHS induced higher PEPCK content and decreased LDH activity (*p* < 0.05), and tended to increase the GCK content (*p* < 0.1), with no impact on the FAS content. With the increased Se supplementation (0.2, 0.4 and 0.6 mg/kg), the PEPCK content tended to decrease, and the GCK content in the group of Se 0.4 mg/kg was close to the control level. The FAS content and LDH activity were not affected by Se supplementation. We further investigated the response of mRNA level of 12 metabolic-related genes to Se in longissimus dorsi muscle under CHS (Figure 5E–G). CHS down-regulated (*p* < 0.05) the mRNA level of *SREBP1* and up-regulated (*p* < 0.05) the expression of *INSR*. Meanwhile, CHS tended to decrease mRNA levels of *ACC1* and *GCK* (*p* < 0.1). Dietary Se supplementation at 0.4 and 0.6 mg/kg restored (*p* < 0.05) mRNA profiles of *SREBP1*, *ACC1* and *INSR*, which were close to the control level. The Se supplementation enhanced mRNA level of *GCK*. Other than that, CHS and dietary Se supplementation failed to have an effect (*p* > 0.05) on the expression of *AMPKα*, *AKT1*, *mTOR*, *4E-BP1*, *FASN*, *PPARG*, *IRS1,* and *PCK2*.

### 3.6. The mRNA Expression of Selenoproteins

**Figure 6:**
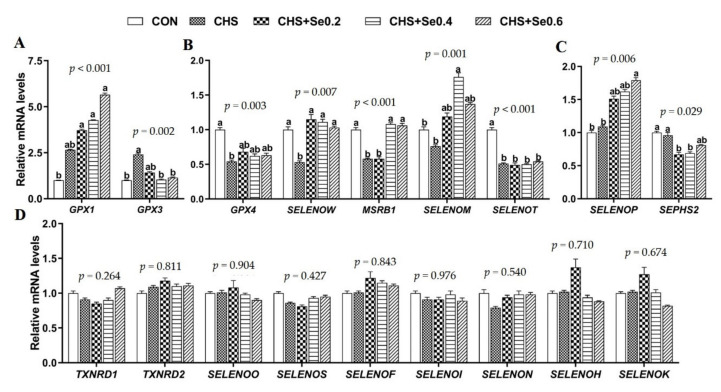
Effects of CHS and OH-SeMet supplementation on expression of selenoprotein encoding genes in longissimus dorsi muscle (**A**–**D**). The results are expressed as Mean ± SEM (*n* = 6). Different letters (a, b, c, d) denote significant differences (*p* < 0.05).

The mRNA abundance of 25 selenoprotein encoding genes in longissimus dorsi muscle were explored (Figure 6). All except for seven selenoprotein encoding genes were too close to the background to be interpreted or reported. CHS up-regulated mRNA expression of *GPX3* (*p* < 0.05) and *GPX1* (*p* < 0.1). Se supplementation tended to increase the mRNA expression of *GPX1*, and the mRNA level of *GPX3* returned to the control level in the groups supplemented with Se 0.4 and 0.6 mg/kg when compared to those of the CHS group (Figure 6A). In addition, CHS down-regulated (*p* < 0.05) the expression of 4 selenoprotein encoding genes (*GPX4*, *SELENOW*, *MSRB1,* and *SELENOT*), and numerically decreased the expression of *SELENOM* (Figure 6B). Se supplementation moderately recovered the mRNA expression of *GPX4*. The mRNA expressions of *SELENOW* and *SELENOM* were recovered or enhanced in three Se addition groups under CHS, and Se 0.4 and 0.6 mg/kg normalized the mRNA expression of *MSRB1* (Figure 6B), while *SELENOT* showed no response to the Se supplementation. Although CHS did not affect the mRNA expression of *SELENOP* and *SEPHS2*, Se supplementation enhanced the expression of *SELENOP* encoding gene and inhibited the expression of *SEPHS2* encoding gene. On the other hand, CHS and Se supplementation did not affect the remaining 16 selenoprotein encoding genes (Figure 6D and Table S1).

### 3.7. The Protein Abundance of Selenoproteins

**Figure 7:**
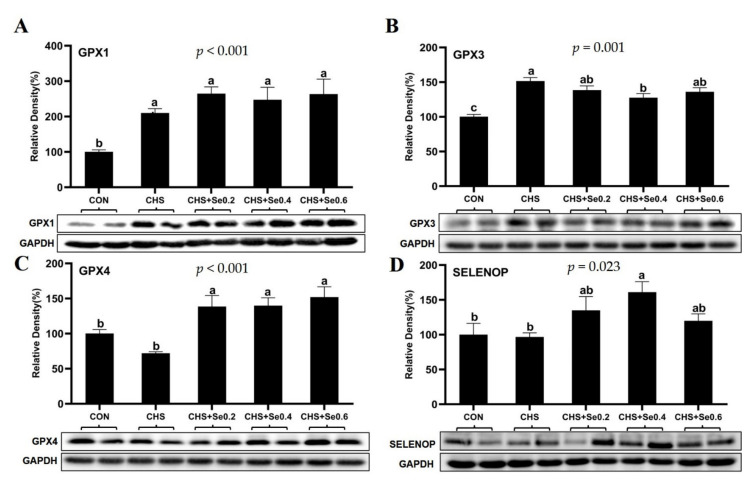
Effects of CHS and OH-SeMet supplementation on protein abundance of GPX1 (**A**), GPX3 (**B**), GPX4 (**C**), and SELENOP (**D**) in longissimus dorsi muscle. The results are expressed as Mean ± SEM (*n* = 6). Different letters (a, b, c, d) denote significant differences (*p* < 0.05).

The effects of CHS and Se supplementation on protein abundance of GPX1, GPX3, GPX4, and SELENOP were investigated (Figure 7). CHS significantly up-regulated (*p* < 0.05) the protein abundance of GPX1 and GPX3, with limited impact on the protein expression of GPX4 and SELENOP. While Se supplementation numerically up-regulated the protein abundance of GPX1, it significantly elevated GPX4 protein abundance (*p* < 0.05). Meanwhile, Se supplementation at 0.4 mg/kg inhibited the GPX3 up-regulation (*p* < 0.05), and enhanced (*p* < 0.05) the protein expression of SELENOP under CHS condition. The Se supplementation at 0.2 and 0.6 mg/kg tended to elevate protein abundance of GPX3 and SELENOP under CHS.

## 4. Discussion

Heat stress, especially chronic heat stress, has been a major environmental constraint for almost all livestock sectors [2]. In the present study, CHS compromised the ADG, ADFI, and final live weight of pigs (Table 2). Reduced feed intake and growth performance is the primary and common response to HS across all species [6,37], which will lead to significant alteration of the carcass traits, as described in Table 3. These results are in line with the findings of lamb and swine [6,7]. It is known that the mechanism of HS or CHS is primarily through over-production of reactive oxygen or nitrogen species (ROS, RNS) that impair the antioxidant capacity at the cellular levels [38]. Therefore, an enforcement of antioxidant capability should protect animal cells and tissues from extensive oxidative damages. Among all known antioxidants, selenium through various selenoproteins plays a key role at cellular levels [39]. As shown in this study, the dietary supplementation with OH-SeMet clearly alleviated the impact of CHS by restoring growth performance, carcass length, carcass weight, and lean proportion, despite not fully restoring these parameters to the level of control without exposure to CHS (Table 3). Our results are well in line with the previous findings that dietary supplementation with antioxidants improves growth performance of poultry, pigs, and sheep under heat stress [7,26,27].

Chronic heat stress also impairs meat quality [5], resulting in pale-soft-exudative (PSE) meat in poultry and pigs [23,24]. In the present study, CHS caused PSE-like pork (Figure 2), mainly manifested by the increased L* and b* values, and the decreased a* value during 45 min to 96 h after slaughtering (Table 4). Previous studies demonstrated that dietary selenium supplementation helped maintain pork colour during storage over time [39,40]. In this study, dietary supplementation with Se 0.4 and 0.6 mg/kg restored the colour of the pork and improved the a* value and inhibited elevation of the L* and b* values 45 min to 96 h after slaughtering. Early studies reported that CHS resulted in decreases in backfat and IMF content in pigs, which may be an adaptive response of the animal in order to achieve better heat dissipation [10,41,42]. Acute heat stress before slaughtering causes rapid decomposition of muscular glycogen, and elevation of lactic acid, decreasing muscular pH and formation of PSE meat of broilers [43]. Yang et al. (2014) reported that long-term heat stress increased the drip loss and shearing force of longissimus muscle of pig [8]. Shakeri et al. (2019) also observed that HS increased drip loss but decreased cooking loss of breast muscle of broiler chicken [44]. Similarly, this study found CHS led to decrease of muscle glycogen content and numerically lower IMF, but increased cooking loss, which may be due to different animal species and slaughtering weight. The supplementation with OH-SeMet buffered the negative impact of the CHS on IMF, drip, and cooking losses. In particular, Se 0.4 and 0.6 mg/kg recovered the muscular glycogen content. Since the CHS damage on meat quality was likely due to oxidative stress [45], selenium plays an important role to prevent myoglobin and lipids from oxidation, subsequently maintaining meat colour [39]. Early studies revealed remarkable correlation between Se deposition and meat quality in different livestock species [34,39,46]. In the present study, while CHS induced elevation of serum and muscle MDA content, and decrease of serum GSH-Px and T-SOD (Table 5), dietary supplementation with OH-SeMet not only effectively elevated Se deposition in the tissues, but the supplementation of Se 0.4 and 0.6 mg/kg also significantly increased GPX-Px activity in the serum and muscle, partly explaining the improvement of the meat quality in this study.

Heat stress, especially chronic heat stress, generally leads to physiological, endocrine, and metabolism perturbations in live animals [9,10], subsequently inducing tissue and organ damages. Among the heat shock proteins (HSPs), HSP70 has been frequently used to evaluate HS damage, thus considered as a cellular thermometer [47]. As expected, in this study CHS increased the LD muscle HSP70 abundance, and the addition of Se 0.4 mg/kg decreased the HSP70 level of LD muscle under CHS (Figure 4E,F).

Acute heat stress activates the hypothalamo-pituitary-adrenal axis, which leads to the release of cortisol [46]. Early studies reported CHS-induced decrease of serum cortisol in pigs [42,48,49]. In the present study, CHS led to significant reduction in serum cortisol concentration (Figure 4A), which may be due to the decreased motion of the pigs. It has been reported that heat stress alters the behavior pattern of pigs, with the pigs spending more time in a resting state [48]. Cortisol is a primary glucocorticoid regulating the glucose and lipid metabolic homeostasis, and activating the immune system [50]. It has been demonstrated that CHS increases CK and LDH concentrations, while reducing ALP concentration in the serum of pigs [51]. The increased serum CK and LDH indicates cardiac, skeletal muscle, and organ injury, and ALP plays an important role in bone development and enteral nutrient absorption [52]. In this study, CHS disturbed the cortisol secretion and increased markers of muscular damage. Certain doses of Se supplementation partially recovered serum cortisol and ALP concentration and moderately decreased the serum CK, LDH concentrations, and sarcous HSP70 abundance, which implies degrees of alleviating skeletal muscular injury and metabolic abnormalities under CHS.

Heat stress directly affects the organ and muscle metabolism of pigs which can continue after slaughtering [11,23], as the metabolism of energy and protein continues with related enzymes in the muscular tissues affecting aspects of meat quality [32,53]. In this study, CHS caused significant down-regulation of mRNA expression of *SREBP1* and up-regulation of the mRNA expression of *INSR*. A trend for decreased mRNA expression of *ACC1* and *GCK* were also observed. FASN, SREBP1, PPARG, and ACC1 are involved in the fatty acid synthesis and lipogenesis, and the expression of these genes are positive to the intramuscular fat deposition [54]. Thus, the lower mRNA expression of *SREBP1* and *ACC1* in LD muscle of CHS pigs may explain the numerically decreased IMF in this study. Our previous study found that CHS decreased the serum insulin [11]. In this study, the up-regulation *INSR* is presumably an adaptive response to decreased circulating insulin. GCK catalyzes the conversion of glucose to glucose 6-phosphate in the cytoplasm which is an important process in glycogen synthesis [55]. Similar to our previous study [11], CHS tended to decrease the mRNA expression of *GCK*, which may result in the lower muscle glycogen content recoded in this study. Interestingly, in the present study, the supplementation with Se 0.4 and 0.6 mg/kg restored the expression of *SREBP1* and *ACC1*, enhanced the *GCK* mRNA level, and normalized the mRNA expression of *INSR*, which may have supported the recovery of muscular IMF and glycogen under CHS. Nevertheless, the expression of *AMPKα1* and protein synthesis-related genes (*AKT1*, *mTOR,* and *4-E-BP1*) were not sensitive to CHS and Se supplementation, which may be related to the hierarchy of maintaining cell survival. PEPCK is an important kinase in gluconeogenesis. Early research demonstrated that increased PEPCK is a common response to environmental stress [56], which converts non-sugar substances (glycerin, lactic acid, and glycogenic amino acids) into glucose to cope with stress [57]. Consistently, in the current study, as the Se supplementation increases, the sarcous PEPCK shows a decreasing trend under CHS. During pig muscle development, LDH activity represents muscle maturity, which was positively correlated with growth performance [58,59]. This study also confirmed that CHS lead to a decrease in LDH activity and growth performance. We noticed the lack of influence of CHS and Se on FAS in mRNA and protein level, and that influence was inconsistent with GCK mRNA expression and protein abundance in the current study (Figure 5). These results may be due to the characteristic of fat deposition of growing stage of the pigs and the complicated regulation in transcription, mRNA decay, and translation [60]. Nonetheless, the Se supplementation at 0.4 and 0.6 mg/kg partially recovered the negative effect of CHS on metabolism-related gene expression and enzyme activity of muscle.

In addition to the antioxidant activity of Se, a previous study [61] demonstrated an important role for Se and certain selenoproteins in muscular metabolism and their implication in muscular diseases. Our previous in vitro and in vivo studies also detected that HS caused unique alternation of selenogenome [11,62]. In this study, we detected 9 selenoprotein encoding genes in muscle that responded to CHS and Se supplementation with 3 distinct patterns observed (Figure 6).

Firstly, CHS led to up-regulation of *GPX1* and *GPX3* mRNA and protein levels (Figure 6A), which was consistent with our previous study in mouse myoblast cells [62]. OH-SeMet supplementation enhanced mRNA and protein expression of GPX1, recovered *GPX3* mRNA expression to the control level (Figure 6A and Figure 7). Since GPX1 and GPX3 belong to the major antioxidant enzyme family GPXs [17], the up-regulation of these two genes suggests the enhanced antioxidant capacity to cope with the oxidative stress caused by hyperthermia. Meanwhile, recent studies in mice have found overexpression of *GPX1* is accompanied by the up-regulation of *GCK*, *PEPCK* and *ACC1* in liver or muscle tissue [63]; this study found similar responses. Previous studies suggested the mRNA expression of *GPX3* was not affected by feeding a high-Se diet [15] and its up-regulation was associated with decreased gene expression of *GCK*, *ACC1,* and *SREBP1* [17]; such negative correlation was also observed in this study (Figure 6A and Figure 7).

The second pattern was that CHS led to down-regulation of *GPX4*, *SELENOW*, *MSRB1*, *SELENOM,* and *SELENOT*. The supplementation with OH-SeMet recovered expression of these selenoprotein encoding genes decreased by CHS, except for *SELENOT* (Figure 6B). GPX4, a member of the GPXs family, is essential for the termination of lipid peroxidation and to reduce phospholipid hydroperoxides [64]. The oxidative stress induced by heat stress is accompanied by the production of lipid peroxides [9], and the mRNA and protein expression of GPX4 in muscle tissue was negatively related to the MDA content in the serum and muscle tissue (Figure 6B and Figure 7C; Table 5). It has been reported that the white muscle disease induced by a low Se diet is associated with the decrease of SELENOW in the calf [61]. Although the exact mechanism has yet to be established, the drip loss of pork is negatively correlated with the mRNA expression of *SELENOW* [34]. On the contrary, in this study, the drip loss trended to increase with the elevated mRNA expression of *SELENOW* (Figure 6B; Table 4). This inconsistency may be due to different slaughtering weight; the precise function of SELENOW in this process is yet to be investigated. MSRB1 is involved in repairing oxidized methionine residues (methionine-R-sulfoxide) in proteins sensitive to stress and Se supplementation. Similar to GPX1, MSRB1 has been shown to act in the Se-mediated regulation of energy metabolism and diabetes risk [17,65]. Although the exact mechanism remains unclear, the mRNA expression of *MSRB1* in response to CHS and Se supplementation was consistent with *SREBP1*, *ACC1,* and *GCK* in this study. SELENOM is an endoplasmic reticulum (ER)-resident protein, which is a thiol-disulfide oxidoreductase and participates in the regulation of apoptosis [66]. Meanwhile, SELENOM in spleen of pigs is sensitive to the dietary Se concentration [67], and is down-regulated in the IPEC-J2 cells HS model [68]. The down-regulation of *SELENOM* in this study may reflect the sarcous oxidative injury induced by CHS. Although the function of SELENOT in porcine remains unclear, its genomics and molecular analysis show its participation in the redox metabolism regulation [69].

The third pattern was that the mRNA expression of *SELENOP* and *SEPSH2* were not affected by CHS, while Se supplementation changed the mRNA or protein expression of SELENOP and *SEPSH2* (Figure 6C; Figure 7D). Recently, several studies have found SELENOP is related to the regulation of insulin signaling and glucose metabolism both in vitro and in vivo [18,69,70,71]. *SEPHS2* encodes the protein of selenophosphate synthetase, which was reported to be insensitive to Se deficiency in rodent tissues [61,72]. However, in this study, Se supplementation down-regulated the mRNA expression of SEPHS2, which may be a negative feedback regulation to the selenoprotein synthesis, and the mechanism needs to be further elucidated. Overall, this study found nine selenoprotein encoding genes (*TXNRD1*, *TXNRD2*, *SELENOO*, *SELENOS*, *SELENOF*, *SELENON*, *SELENOH,* and *SELENOK*) insensitive to CHS and Se supplementation, which may relate to being organ or tissue-specific and hierarchy of selenoprotein under CHS and Se level.

## 5. Conclusions

In conclusion, this study confirmed that CHS significantly compromised growth performance, carcass traits, and meat quality of growing pigs. These abnormal phenotypes were associated with the inhibition of antioxidant defense system, alternation in metabolic and endocrine parameters of serum and muscle, and the aberrant expression of several selenoprotein encoding genes and proteins. Se supplementation in the form of OH-SeMet partly alleviated the CHS-induced negative impacts, especially for meat quality parameters. These mitigations are realized through restoration of metabolic-related mechanism, in particular, the function of selenoproteins-based antioxidant system following effective dietary supplementation with OH-SeMet.

## Figures and Tables

**Table 1:** Composition and nutrients of the basal diets.

Ingredients	% On Feed Basis
Corn grain	76.20
Soybean oil	2.80
Soybean meal	13.00
Wheat bran	4.00
Fishmeal (CP 62.5%)	1.40
L-Lysine·hydrochloride	0.42
DL-Methionine	0.12
L-Threonine	0.14
L-Tryptophan	0.04
Choline chloride 50%	0.10
Calcium carbonate	0.85
Calcium hydrophosphate	0.65
Sodium chloride	0.18
Premix ^a^	0.10
Total	100.00
Nutrient levels ^b^	
Digestible energy (Mcal/kg)	3.40
Crude protein (%)	13.74
Calcium (%)	0.59
STTD Phosphorus (%)	0.28
SID Lysine (%)	0.85
SID Met + Cys (%)	0.48
SID Thr (%)	0.52
SID Trp (%)	0.15

^a^ Premix provided per kilogram of diet: Fe, 50 mg; Cu, 3.5 mg; Zn, 50 mg; Mn, 2 mg; I, 0.14 mg; vitamin A, 1300 IU; vitamin D3, 150 IU; vitamin E, 11 mg; vitamin K3, 0.5 mg; vitamin B2 2 mg; vitamin B6, 1 mg; vitamin B12, 5 μg; pantothenic acid, 7 mg; folic acid, 0.3 mg; biotin 0.05 mg. ^b^ Nutrient levels were calculated values.

**Table 2:** Effects of CHS and OH-SeMet supplementation on growth performance of growing pigs.

	CON	CHS	CHS + Se0.2	CHS + Se0.4	CHS + Se0.6	*p*-Value
LW, kg						
Day 0	49.65 ± 0.68	49.54 ± 1.20	49.50 ± 0.87	49.84 ± 1.02	49.64 ± 2.24	0.999
Day 28	77.06 ± 1.28	71.13 ± 1.80	71.29 ± 1.46	72.69 ± 1.65	72.13 ± 1.33	0.054
ADG, g	945 ± 49 ^a^	731 ± 41 ^b^	760 ± 37 ^b^	782 ± 30 ^b^	773 ± 45 ^b^	0.006
ADFI, g	2572 ± 137 ^a^	2080 ± 119 ^b^	2070 ± 101 ^b^	2007 ± 64 ^b^	2078 ± 155 ^b^	0.010
FCR	2.72 ± 0.04	2.86 ± 0.09	2.73 ± 0.05	2.58 ± 0.08	2.69 ± 0.09	0.152

LW, live weight; ADG, average daily gain; ADFI, average daily feed intake; FCR, feed conversion ratio. Data are shown as means ± SEM, *n* = 8 in each group. CON, control group fed on basal diet; CHS, chronic heat stress group fed on basal diet; CHS + Se 0.2, 0.4 and 0.6, CHS supplemented with Se in mg/kg; ^a, b^ Means within the same row not bearing the same superscript alphabet letter differ significantly (*p* < 0.05).

**Table 3:** Effects of CHS and OH-SeMet supplementation on carcass traits of growing pigs.

	CON	CHS	CHS + Se0.2	CHS + Se0.4	CHS + Se0.6	*p*-Value
Carcass length (cm)	89.07 ± 1.25 ^a^	84.37 ± 1.49 ^b^	87.93 ± 1.46 ^ab^	84.73 ± 0.81 ^b^	84.63 ± 0.97 ^b^	0.030
Carcass weight (kg)	54.53 ± 1.09 ^a^	47.96 ± 0.83 ^b^	50.56 ± 1.31 ^ab^	50.63 ± 1.75 ^ab^	51.76 ± 1.13 ^ab^	0.020
Kill-out (%)	69.86 ± 0.83	69.70 ± 0.62	71.28 ± 0.49	68.68 ± 0.95	70.42 ± 0.82	0.207
Lean proportion (%)	48.63 ± 0.35 ^a^	41.50 ± 1.38 ^b^	42.57 ± 1.35 ^b^	44.23 ± 1.41 ^ab^	42.25 ± 0.55 ^b^	0.001
Abdominal fat (%)	0.96 ± 0.08	0.73 ± 0.12	0.83 ± 0.14	0.69 ± 0.11	0.72 ± 0.11	0.447
Backfat (mm)	20.11 ± 1.14	18.26 ± 1.63	18.16 ± 0.35	17.72 ± 1.18	19.84 ± 0.31	0.403
Eye-muscle area (cm^2^)	53.96 ± 2.23	50.74 ± 2.38	49.82 ± 2.17	54.35 ± 1.80	52.67 ± 1.89	0.711

Data are expressed as Means ± SEM, *n* = 6 in each group. CON, control group fed a basal diet; CHS, chronic heat stress group fed a basal diet; CHS+ Se 0.2, 0.4 and 0.6 mg/kg; ^a, b^ Means in the same row not bearing the same superscript alphabet letters differ significantly (*p* < 0.05).

**Table 4:** Effects of CHS and OH-SeMet supplementation on meat quality of pigs.

L*	CON	CHS	CHS + Se0.2	CHS + Se0.4	CHS + Se0.6	*p*-Value
L*_45 min_	44.65 ± 0.56 ^bc^	49.45 ± 0.55 ^a^	49.62 ± 1.09 ^a^	45.94 ± 0.51 ^bc^	48.53 ± 0.60 ^ab^	<0.001
L*_24 h_	55.79 ± 0.80 ^b^	58.62 ± 0.83 ^ab^	59.68 ± 0.99 ^a^	56.59 ± 1.00 ^ab^	57.22 ± 0.83 ^ab^	0.034
L*_48 h_	54.84 ± 0.59 ^c^	61.03 ± 0.86 ^a^	59.15 ± 0.75 ^ab^	58.76 ± 0.36 ^ab^	57.98 ± 0.66 ^b^	<0.001
L*_72 h_	56.92 ± 0.65 ^b^	61.84 ± 1.21 ^a^	61.19 ± 0.76 ^a^	58.48 ± 0.80 ^ab^	58.59 ± 0.69 ^ab^	0.002
L*_96 h_	56.43 ± 0.97 ^b^	62.22 ± 1.25 ^a^	61.98 ± 0.68 ^a^	58.33 ± 1.14 ^ab^	59.28 ± 0.53 ^ab^	<0.001
a*						
a*_45 min_	6.66 ± 0.31	6.52 ± 0.23	6.41 ± 0.10	7.38 ± 0.15	6.89 ± 0.36	0.169
a *_24 h_	10.38 ± 0.58 ^ab^	9.71 ± 0.24 ^a^	9.55 ± 0.42 ^a^	11.62 ± 0.40 ^b^	11.36 ± 0.42 ^b^	0.005
a*_48 h_	9.86 ± 0.46 ^bc^	9.19 ± 0.14 ^c^	10.05 ± 0.37 ^bc^	11.78 ± 0.29 ^a^	10.99 ± 0.95 ^ab^	<0.001
a*_72 h_	9.27 ± 0.52 ^bc^	9.69 ± 0.10 ^b^	8.62 ± 0.27 ^c^	10.28 ± 0.24 ^ab^	10.79 ± 0.33 ^a^	0.001
a*_96 h_	9.84 ± 0.57 ^ab^	8.66 ± 0.17 ^a^	9.10 ± 0.46 ^ab^	10.55 ± 0.29 ^b^	10.65 ± 0.36 ^b^	0.005
b*						
b*_45 min_	5.46 ± 0.17 ^b^	6.88 ± 0.34 ^a^	6.64 ± 0.33 ^ab^	6.10 ± 0.35 ^ab^	5.78 ± 0.21 ^ab^	0.011
b*_24 h_	7.42 ± 0.22 ^b^	9.19 ± 0.43 ^a^	8.39 ± 0.26 ^ab^	8.11 ± 0.28 ^ab^	8.23 ± 0.23 ^ab^	0.006
b*_48 h_	7.47 ± 0.34	8.65 ± 0.43	8.52 ± 0.32	8.25 ± 0.33	8.06 ± 0.29	0.164
b*_72 h_	7.17 ± 0.18 ^b^	8.60 ± 0.39 ^a^	8.47 ± 0.39 ^a^	7.54 ± 0.26 ^ab^	7.93 ± 0.19 ^ab^	0.010
b*_96 h_	7.47 ± 0.28	8.61 ± 0.60	8.55 ± 0.32	7.80 ± 0.30	7.89 ± 0.15	0.108
pH						
pH_45 min_	6.66 ± 0.06	6.53 ± 0.08	6.69 ± 0.08	6.50 ± 0.09	6.51 ± 0.09	0.280
pH_24 h_	5.54 ± 0.01	5.54 ± 0.01	5.59 ± 0.02	5.56 ± 0.01	5.57 ± 0.02	0.116
pH_48 h_	5.57 ± 0.01	5.55 ± 0.02	5.57 ± 0.02	5.58 ± 0.02	5.57 ± 0.02	0.829
pH_72 h_	5.55 ± 0.01	5.58 ± 0.02	5.62 ± 0.01	5.60 ± 0.03	5.60 ± 0.02	0.054
pH_96 h_	5.57 ± 0.01	5.62 ± 0.01	5.63 ± 0.01	5.63 ± 0.03	5.63 ± 0.02	0.218
IMF (%)	3.70 ± 0.37	2.73 ± 0.15	3.93 ± 0.44	3.41 ± 0.34	3.29 ± 0.31	0.167
Glycogen, mg/g	3.49 ± 0.46 ^a^	1.57 ± 0.33 ^b^	1.63 ± 0.29 ^b^	3.06 ± 0.20 ^a^	2.96 ± 0.53 ^a^	0.002
Peak shear force, kg	2.30 ± 0.17	2.24 ± 0.19	2.06 ± 0.10	2.02 ± 0.23	2.49 ± 0.14	0.351
Drip loss %	3.14 ± 0.16	2.52 ± 0.14	2.68 ± 0.23	3.82 ± 0.56	3.94 ± 0.66	0.064
Cooking loss %	30.39 ± 0.83 ^b^	37.26 ± 0.23 ^a^	33.45 ± 0.68 ^ab^	34.83 ± 1.17 ^ab^	34.19 ± 1.51 ^ab^	0.005

Data are expressed as Means ± SEM (*n* = 6). CON, control group fed on basal diet; CHS, chronic heat stress group fed on basal diet; CHS + Se 0.2, 0.4 and 0.6 mg/kg; ^a, b, c^ Means within the same row not bearing the same superscript letters differ significantly (*p* < 0.05). L*, lightness; a*, redness; b*, yellowness; IMF, intramuscular fat.

**Table 5:** Effects of CHS and OH-SeMet supplementation on serum and muscle antioxidant enzyme and malondialdehyde content of pigs.

Parameters	CON	CHS	CHS + Se0.2	CHS + Se0.4	CHS + Se0.6	*p*-Value
Serum						
GSH-Px, U/mL	582 ± 22 ^b^	581 ± 47 ^b^	929 ± 23 ^a^	943 ± 57 ^a^	987 ± 45 ^a^	<0.001
MDA, nmol/mL	2.43 ± 0.16 ^b^	4.00 ± 0.56 ^a^	2.87 ± 0.34 ^ab^	2.24 ± 0.14 ^b^	2.79 ± 0.15 ^ab^	0.012
T-SOD, U/mL	234 ± 20 ^a^	169 ± 25 ^b^	169 ± 23 ^b^	212 ± 27 ^ab^	178 ± 20 ^ab^	0.010
T-AOC, U/mL	2.71 ± 0.07 ^ab^	2.21 ± 0.13 ^b^	3.20 ± 0.30 ^a^	3.47 ± 0.37 ^a^	3.36 ± 0.65 ^ab^	0.011
Muscle						
GSH-Px, U/mg prot	3.99 ± 0.61 ^b^	4.15 ± 0.36 ^b^	4.30 ± 0.54 ^ab^	6.47 ± 0.87 ^a^	6.01 ± 0.20 ^a^	0.009
MDA, nmol/mg prot	0.79 ± 0.08 ^b^	1.32 ± 0.16 ^a^	0.73 ± 0.05 ^b^	0.75 ± 0.04 ^b^	0.88 ± 0.08 ^b^	0.001
T-SOD, U/mg prot	0.93 ± 0.04	1.08 ± 0.10	1.00 ± 0.04	1.05 ± 0.09	1.00 ± 0.05	0.636
T-AOC, U/mg prot	0.13 ± 0.02	0.09 ± 0.01	0.12 ± 0.01	0.13 ± 0.02	0.15 ± 0.03	0.467

GSH-Px, glutathione peroxidase; MDA, malondialdehyde; T-SOD, total superoxide dismutase; T-AOC, total antioxidant capability. The results were expressed as Mean ± SEM (*n* = 6). ^a, b^ Values within a row not bearing the same superscripts differ (*p* < 0.05).

## Data Availability

The data presented in this study are available in the article and supplementary material.

## Supplementary Materials

The following are available online at https://www.mdpi.com/article/10.3390/antiox10101558/s1, Table S1: Primers used for the q-PCR of the target and reference genes.
