# Supplementary material for: Hydroxy Selenomethionine Improves Meat Quality through Optimal Skeletal Metabolism and Functions of Selenoproteins of Pigs under Chronic Heat Stress
Source: Antioxidants (Basel). 2021 Sep 29;10(10):1558. doi: 10.3390/antiox10101558 (PMC8533020; doi:10.3390/antiox10101558)
Supplement: Supplementary file 1 [file antioxidants-10-01558-s001.zip › antioxidants-1392599-supplementary.pdf]

Supporting Table S1. Primers used for the q-PCR of the target and reference genes

Protein	Gene	Accession number	Primer sequence (from 5' to 3')
		**Selenoprotein encoding genes**	
DIO1	*DIO1*	AY533206	F: CATGGCCAAGAACCCTCACT<br>R: CCAGAAATACTGGGCACTGAAGA
DIO2	*DIO2*	AY533207	F: CGCTGCATCTGGAAGAGCTT<br>R: TGGAATTGGGTGCATCTTCA
DIO3	*DIO3*	AY533208	F: TGAAGTGGAGCTCAACAGTGATG<br>R: TGTCGTCAGACACGCAGATAGG
GPX1	*GPX1*	AF532927	F: GATGCCACTGCCCTCATGA<br>R: TCGAAGTTCCATGCGATGTC
GPX2	*GPX2*	DQ898282	F: AGAATGTGGCCTCGCTCTGA<br>R: GGCATTGCAGCTCGTTGAG
GPX3	*GPX3*	AY368622	F: CCGGTTCCTGTTTTCCAAATT<br>R: TGCACTGCAGGAAGAGTTTGAA
GPX4	*GPX4*	NM_214407	F: TGAGGCAAGACGGAGGTAAACT<br>R: TCCGTAAACCACACTCAGCATATC
GPX6	*GPX6*	NM_001137607	F: GAGCTGAAGCCTTTTGGTGTAGTT<br>R: CTTTGCTGGTTCTTGTTTTCCA
MSRB1	*MSRB1*	EF113597	F: ATCCCTAAAGGCCAAGAATCATC<br>R: GGCCACCAAGCAGTGTTCA
SELENOF	*SELENOF*	EF178474	F: ACAGCCCTGCCAAGCAGAT<br>R: AACAGGGAGGCTGGGTAACAC
SELENOH	*SELENOH*	HM018602	F: TGGTGGAGGAGCTGAAGAAGTAC<br>R: CGTCATAAATGCTCCAACATCAC
SELENOI	*SELENOI*	NM_001244662.1	F: GATGGTGTGGATGGAAAGCAA<br>R: GCCATGGTCAAAGAGTTCTCCTA
SELENOK	*SELENOK*	DQ372075	F: CAGGAAACCCCCCTAGAAGAA<br>R: CTCATCCACCGGCCATTG

SELENOM	*SELENOM*	FJ968780	F: CAGCTGAATCGCCTCAAAGAG<br>R: GAGATGTTTCATGACCAGGTTGTG
SELENON	*SELENON*	EF113595	F: ACCTGGTCCCTGGTGAAAGAG<br>R: AGGCCAGCCAGCTTCTTGT
SELENOO	*SELENOO*	AK236851	F: CTTCCGACCCCAGATGGAT<br>R: GGTTCGACTGTGCCAGCAT
SELENOP	*SELENOP*	EF113596	F: AACCAGAAGCGCCAGACACT<br>R: TGCTGGCATATCTCAGTTCTCAGA
SELENOS	*SELENOS*	AY609646	F: GAGGCAGAGGCACCTGGAT<br>R: CTGCTAAAGCCTCCTGTCGTTT
SELENOT	*SELENOT*	AY609428	F: GGCTTAATAATCGTTGGCAAAGA<br>R: TGGCCCCATTGCCAGATA
SELENOV	*SELENOV*	GQ478346	F: CACTGGTCGCCAATGGATTC<br>R: AGTGGCCAACGGAGAAAGC
SELENOW	*SELENOW*	NM_213977	F: CACCCCTGTCTCCCTGCAT<br>R: GAGCAGGATCACCCCAAACA
SEPHS2	*SEPHS2*	EF033624	F: TGGCTTGATGCACACGTTTAA<br>R: TGCGAGTGTCCCAGAATGC
TXNRD1	*TXNRD1*	AF537300	F: GATTTAACAAGCGGGTCATGGT<br>R: CAACCTACATTCACACACGTTCCT
TXNRD2	*TXNRD2*	GU181287	F: TCTTGAAAGGCGGAAAAGAGAT<br>R: TCGGTCGCCCTCCAGTAG
TXNRD3	*TXNRD3*	BX918808	F: GTGCCCTACGTTTATGCTGTTG<br>R: TCCGAGCCACCAGCTTTG
**Metabolism-related genes**			
AMPKα1	*AMPKα1*	NM_001167633.1	F: TTGACTCGGCCCCATCCT<br>R: GTATGGCGTGCCCTTGGA
GCK	*GCK*	XM_013985832.2	F: GTGGTGGCAATGGTGAATGAC<br>R: TCGGCGGTCTTCATAGTAGCA

PCK2	*PCK2*	NM_001161753.1	F: GCCCTTCTTCGGCTACAACTTT<br>R: CCTTGCGCCCCTCCAT
INSR	*INSR*	AF102858.1	F: CCAAAGGCCAGCCAACACT<br>R: GGGAACGCAGGTAACTCTTTAAGTC
AKT1	*AKT1*	NM_001159776.1	F: AGAACCGCGTCCTCCAGAA<br>R: CGTGGGTCTGGAAGGAGTACTTC
SREBF1	*SREBF1*	NM_214157.1	F: GCTGAATAAATCCGCCGTCTT<br>R: CTGGTTGCTCTGCTGAAGGAA
ACC1	*ACC1*	XM_021066238.1	F: CAAGACCACCAACGCGAAA<br>R: GGCAAATGGGAGGCAATAAGA
PPARG	*PPARG*	NM_214379.1	F: TGCCACAGGCTGAGAAGGA<br>R: GGGTTCAGCTGGTCGATATCAC
FASN	*FASN*	NM_001099930.1	F: GTGGGTGTGAGCAGTTCTGATG<br>R: GCCCCTTGAAGTCAAAGAAGAAG
mTOR	*mTOR*	XM_003127584.6	F: GGACACAAACACCAAAGGTAACAAG<br>R: GTGGTCCCCGTTTTCTTATGG
4E-BP1	*4E-BP1*	NM_001244225.1	F: CCCCCTGCTTCCTCACTCA<br>R: TGGAGGTATCTGCTGGTGTTCA
**Housekeeping control genes**			
β-ACTIN	*β-ACTIN*	NM_007393.5	F: ACCAGTTCGCCATGGATGAC<br>R: TGCCGGAGCCGTTGTC
GAPDH	*GAPDH*	GU214026.1	F: GGGAAGCCCATCACCATCT<br>R: CGGCCTCACCCCATTTG
